# Assault‐on‐Law‐Enforcement: The Complications of Charges for Individuals on Mental Health Court Dockets

**DOI:** 10.1002/cbm.70048

**Published:** 2026-09-03

**Authors:** Steven Keener, Audrey Morrison

**Affiliations:** ^1^ Department of Sociology, Social Work, and Anthropology Christopher Newport University Newport News Virginia USA

**Keywords:** diversion, mandatory minimums, mental health, problem‐solving courts

## Abstract

**Background:**

Mental health courts divert individuals with serious mental illness away from imprisonment into community‐based treatment and supervision. Early research indicates that such treatment contracts can reduce recidivism and provide various other benefits. Aspects of eligibility for this diversion option are still under‐researched.

**Aims:**

To explore the nature of alleged offences against people accepted into mental health courts (on mental health court dockets), and their demographic and mental health context and their perceived relevance to eligibility for such diversion.

**Methods:**

We used a sequential mixed‐methods design in which semi‐structured interviews were conducted after completion of quantitative data collection from the State of Virginia's behavioural and therapeutic court docket cases in 2022–2023. The State provides open online data on demographics and the nature of alleged offences for all mental health court‐adjudicated cases. The qualitative part of the study involved semi‐structured interviews with behavioural health docket coordinators across Virginia about their perceptions of how the nature of alleged criminal offences affects potential mental health court participation and the dynamics with other disciplines in the system that may be relevant to decision‐making here.

**Results:**

The most commonly alleged offence was assault‐on‐law‐enforcement officers, an offence that, if convicted, carries a mandatory prison sentence. This was disproportionately charged against Black men. Qualitative findings revealed that the coordinators viewed assault‐on‐law‐enforcement officers as likely to have occurred when defendants were in a state of mental health crisis, with resultant perceptions of law enforcement as a threat, and thus, usually, a mental health solution was likely to be in the best interests of all parties. There was local variation in processing, but a sense that local prosecutors hold disproportionate power, and defence lawyers are constrained by ideological advice rather than knowledge of optimal outcomes for their client.

**Conclusions:**

The punitive 6‐month mandatory minimum jail term that must follow a conviction for an assault‐on‐law‐enforcement officer may put both justice and safety at risk. Black men are disproportionately likely to be subject to such allegations, suggesting that disadvantage at every level of health and justice has reached this point. When mental disorder is manifestly a relevant factor in alleged offending, it makes sense to order disposals that will maximise restoration of health and social functioning in order to safeguard such officers, the wider community and the alleged offender. At present, the structured diversion option is only open in Virginia when a mental health court is willing to accept violent felony charges. We recommend wider dissemination of data on mental health court outcomes, including longer‐term outcomes, to law enforcers and politicians with a view to change in the relevant law.

## 1 Introduction

Mental health courts are a growing option for the judiciary. These courts allow diversion from justice into health in appropriate cases, with monitoring to ensure compliance with a treatment plan that connects them to community mental health resources and reduces or drops charges upon successful completion (Belenko 1998; Lurigio and Snowden 2009). Early research indicates that mental health courts can reduce recidivism risks (Fox et al. 2021; Loong et al. 2019). With this early success, it is important to understand who qualifies for diversion. In this study, we used a mixed‐methods approach to analyse the role of alleged offences in determining mental health diversion eligibility.

Research into types of offences adjudicated by a mental health court is important because the first stage filters out participants based upon criminal charges (Wolff et al. 2011). Historically, diversion courts excluded people under violence charges; however, drug courts, which led the way and served as a model for mental health courts, eventually expanded admission to include more serious charges (Belenko 1998; Saum et al. 2001). In the 2022–24 Virginia behavioural health docket annual reports, assault, battery and wounding were treated as, in effect, one offence category and was the most common on the dockets (Supreme Court of Virginia 2022, 2023, 2024). V. A. Hiday and Ray (2010) noted that in the mental health court they studied, approximately 90% of the offences adjudicated were misdemeanours. Of the 10% of felony offences, most were theft. Ray et al. (2015) found, in a statewide database of mental health court defendants, that 41% of defendants had a felony target arrest, suggesting that as these courts became well established, confidence grew about who could be safely managed this way and court actors became bolder in allowing defendants with higher‐level alleged offences this opportunity. Some veteran court dockets accept violent offences, but others do not (Flatley et al. 2017), and the most common veteran docket charges remain public order offences, driving under the influence and drug offences (Tsai et al. 2018).

Evaluations of mental health courts have found generally positive outcomes, a consistent reduction in criminogenic risks and reduced rearrests (Cosden et al. 2003; A. Hiday et al. 2015; Moore and Hiday 2006; Rossman et al. 2012; Steadman et al. 2011; Trupin and Richards 2003; Yuan and Capriotti 2019). This is especially true when individuals connect to housing and employment (Fox et al. 2021; Jalain et al. 2024; Loong et al. 2019). Effectiveness is shaped by the quality of community mental health services, court team staffing levels and interactions with the judge (Boothroyd et al. 2005; Frailing 2010; Morin 2004). Remaining concerns exist about defendants who may not be competent to enter into such an agreement with the courts but could benefit from such arrangements (Erickson et al. 2006). Early indications are, thus, that mental health courts can reduce recidivism risks and help participants, so it is important to know if this option is yet widely available to people who could benefit but are charged with offences that are above the misdemeanour level and, if not, the nature of the barriers.

In this study, therefore, our aim was to answer the following questions: (1) What types of charges are current mental health docket participants most commonly facing? (2) Do the types of charges vary across demographic characteristics? (3) How do individuals on mental health courts incur these charges? (4) How do mental health court teams decide who is eligible to take part on the docket?

## 2 Methods

### 2.1 Ethics

The study was approved by the Christopher Newport University Institutional Review Board.

### 2.2 Design and Sampling

We implemented an explanatory sequential mixed‐methods design, in which we collected and analysed the quantitative data before beginning qualitative data collection (Edmonds and Kennedy 2017).

All individuals whose cases were on record as diverted onto the behavioural health or therapeutic dockets between January 2022 and December 2023 were included; the only potential source of error here was an input error on the court website, but we found no evidence of such an error. Publicly available information on Virginia's Court System website was used to build the quantitative dataset (General District Court, n.d.). In Virginia, mental health courts are called ‘behavioural health or therapeutic dockets’. We used a state directory to identify the localities that had a behavioural health or therapeutic court docket and when they met. We then searched the cases heard on the days and times that each docket met and recorded details of the cases throughout the complete years 2022–2023. We recorded the sex and race of the defendant, the locality of the case and charges in a spreadsheet. There is no directory of all cases that were considered but not diverted, so we relied on the qualitative methodology to learn about why some cases are accepted onto the docket but others are not.

We collected the qualitative data through semi‐structured interviews with behavioural health and therapeutic docket coordinators across Virginia. We interviewed docket coordinators because they oversee all operations of the docket, ranging from the justice system to therapeutic requirements. A pretrial officer on the docket team may work with a participant on their justice system requirements, but then a case manager may work with them on meeting with their mental health providers. Coordinators oversee all elements of these participant responsibilities while also working daily with behavioural health and therapeutic docket staff. The interview guide had questions on coordinators' backgrounds, court participant charges, factors leading to those charges and how they make decisions about who qualifies for the docket. The questions about coordinator background were important because there are no standard educational and/or work experience requirements for this position.

We invited all 20 of the docket coordinators in Virginia at the time of data collection to take part. Ten coordinators ultimately participated in an interview on Google Meet for approximately 1 hour between May and August 2024. Participants provided consent for the interview and for it to be recorded.

### 2.3 Planned Analyses

Docket case data were analysed with SPSS using a series of basic descriptive and bivariate analysis techniques.

We transcribed each interview using Otter.ai and checked the transcripts manually. We then analysed the interview transcripts in ATLAS.ti to find thematic patterns and conceptualise how the results informed the quantitative results. ATLAS.ti is an important and useful tool for qualitative data analysis that helps with coding and analysis, creating a transparent and rigorous documentation of the steps taken. This was especially helpful in finding important and impactful quotes from participants that illuminated the thematic results (Paulus and Lester 2016; Scales 2013).

## 3 Results

### 3.1 Range of Offences as Charged in Virginia's Behavioural Health and Therapeutic Courts During 2022–23

Figure 1 shows the distribution of alleged offences under consideration. It is important to stress that these are offences, not individuals, some of whom may have had several recorded. By far the most commonly occurring charge over the 24 months studied in Virginia's behavioural health and therapeutic court dockets was assault‐on‐law‐enforcement officers (*n* = 376, 12%), with trespassing (*n* = 247, 8%) and assault and battery (*n* = 143, 5%) also quite common. Just three other charge types crossed the 100 threshold—destruction of property worth less than $1000 with intent (*n* = 104, 3%), possession of a Schedule I or II substance (*n* = 103, 3%) and petty larceny worth less than $1000 not from a person (*n* = 100, 3%).

**FIGURE 1:**
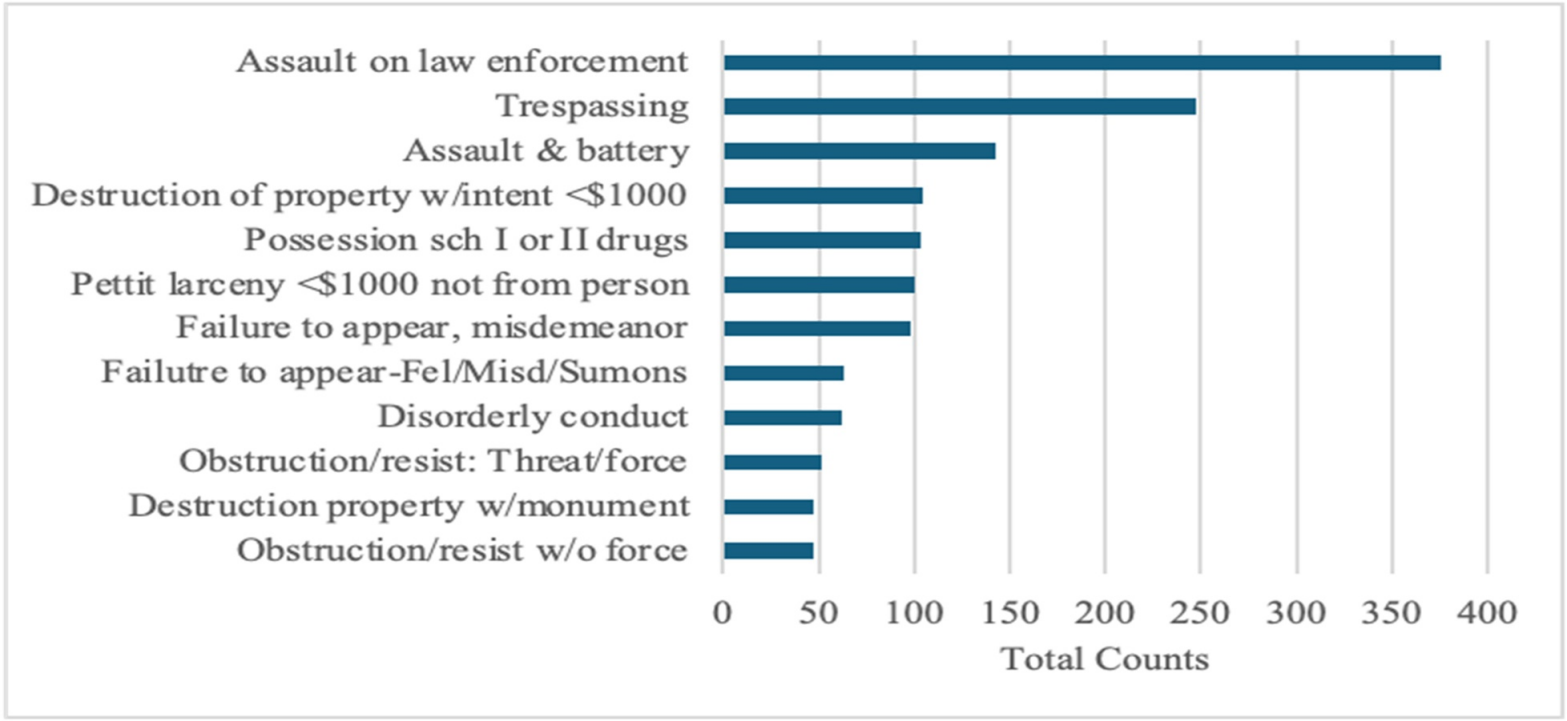
Total counts of most common charges on Virginia's mental health courts, 2022–2023.

The next analysis focused on the individuals. Of the 376 individuals facing an assault‐on‐law‐enforcement charge, nearly half (*n*, 48%) were facing multiple counts of that charge. It was rather less likely to find multiple counts of other charges. With respect to trespassing, for example, less than one third of those charged with trespass had more than one such charge (*n* = 41, 30%), and only 11% (*n* = 10) of those charged with assault and battery were facing multiple counts of it. Also, there were 23 (23%) total individuals with at least one assault‐on‐law‐enforcement charge and a trespassing charge, 14 (14%) total individuals with at least one assault‐on‐law‐enforcement charge and an assault and battery charge, 14 (14%) total individuals with at least one assault‐on‐law‐enforcement charge and a destruction of property charge, 4 (4%) total individuals with at least one assault‐on‐law‐enforcement charge and a possession of a Schedule I or II substance and 3 (3%) total individuals with at least one assault‐on‐law‐enforcement charge and a petty larceny charge. Figure 2 illustrates this graphically.

**FIGURE 2:**
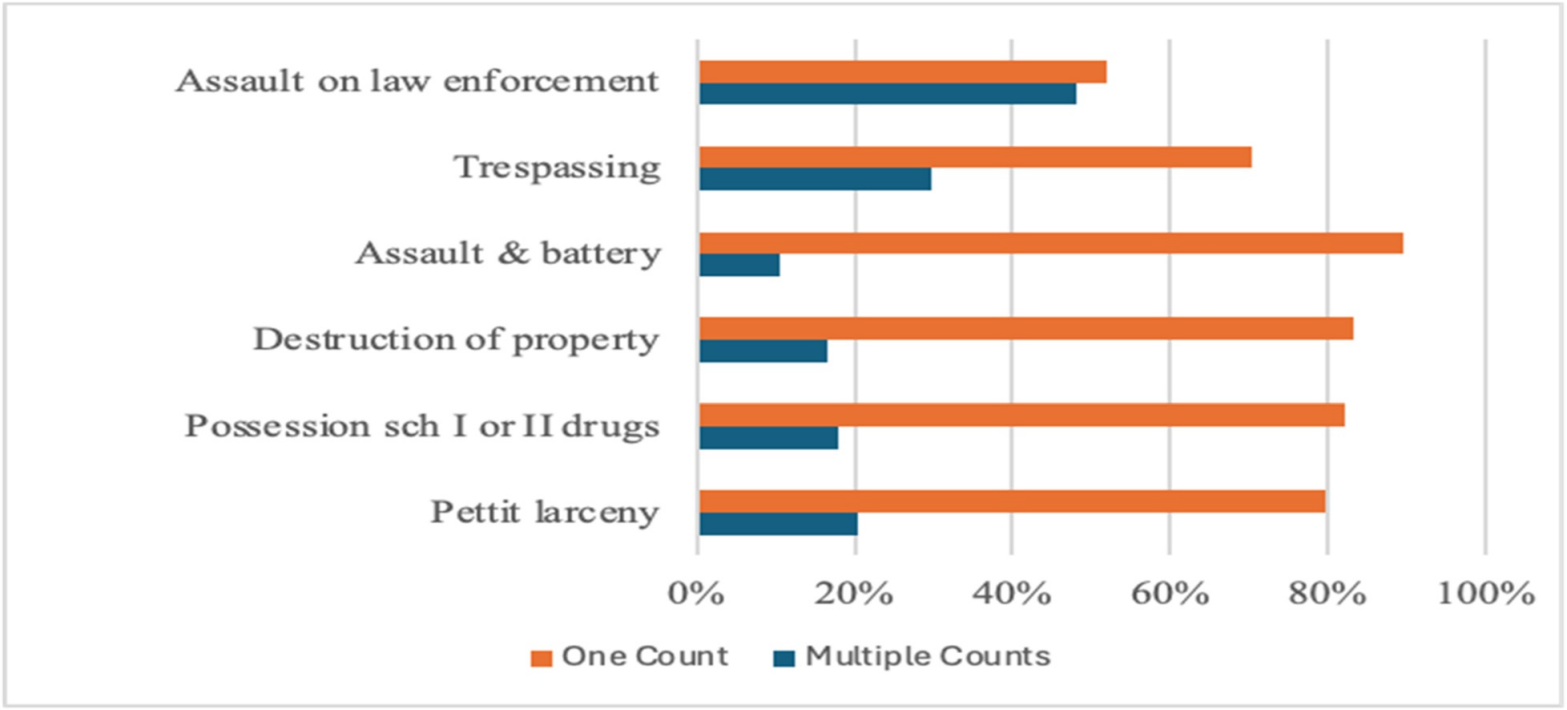
Rate of single versus multiple counts of most common charges in Virginia's mental health courts, 2022–2023. The figure displays, of all the individuals charged with each criminal charge, the rate of those who have one count of that charge and the rate of individuals who have multiple counts of that charge.

Assault‐on‐law‐enforcement officer in Virginia carries a 6‐month mandatory minimum sentence of imprisonment (McCausland and Elders 2020). Given this punitive sentence, we analysed how the rate of individuals charged with this offence varied by race and sex. Of all individuals facing multiple counts of assault against law enforcement officer charges, approximately 47% were Black males, 22% were Black females, 17% were White males, and 14% were White females. Of all individuals facing one count of assault against law enforcement charge, *n* = 31 (31%) were Black men, *n* = 27 (27%) were White men, *n* = 22 (22%) were Black women and *n* = 16 (16%) were White women. Each count for which a defendant is found guilty of assault‐on‐law‐enforcement charges initiates the mandatory 6‐month carceral punishment, so a defendant found guilty of two counts would be facing 12 months behind bars, whereas a defendant found guilty of three counts would be facing 18 months behind bars and so on. If these individuals are not offered an opportunity to join a mental health court and are found guilty, they will be facing these lengthy carceral punishments, whereas those who complete the mental health court are not subject to these mandatory carceral punishments, even if they admit guilt. Figure 3 displays this in more detail.

**FIGURE 3:**
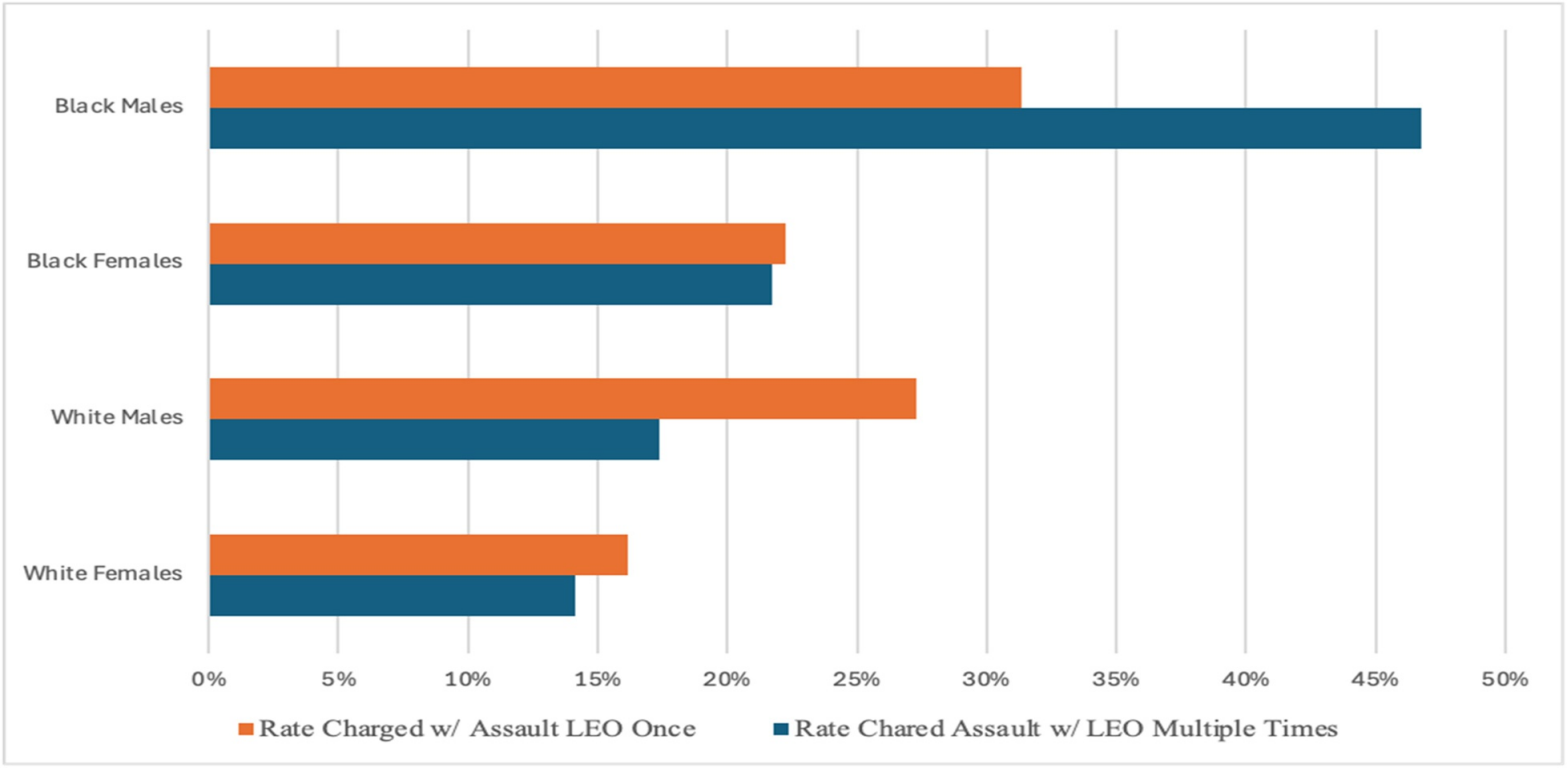
Rate of assault‐on‐law‐enforcement officer charge counts by race and sex among people subject to Virginia's mental health courts 2022–2023. The rates displayed in this figure show the rate of all individuals facing assault‐on‐law‐enforcement charges and how they break down by race and sex.

### 3.2 Interview With Supervisors—The Qualitative Findings

Four primary themes emerged from the qualitative interviews: (1) Assault‐on‐law‐enforcement Charge Dynamics; (2) Legal Requirements of Docket Inclusion; (3) Mental Health and Substance Use Diagnoses; and (4) The Power of Prosecutors in Docket Admission Decisions.

#### 3.2.1 Assault‐on‐Law‐Enforcement Personnel Charge Dynamics

Interview participants typically cited serious mental illness (SMI) as a precipitating factor in assaults when an officer had approached and been seen as a threat; the affected person was seen as reacting defensively, at least in their own mind. One coordinator described, ‘if in the course of that arrest, they elbow the officer, spit on the officer, whatever, they get charged with assault on LEO [law enforcement officer]’. They emphasised that little contact could incur the charge and most officers are not seriously injured. Another coordinator said about the defendants, ‘They're not more likely to be violent, necessarily, but certainly in an escalated state. And if you have officers or community members that don't know how to approach that appropriately, then you know, things get escalated, it gets escalated very quickly’.

In other situations, the incident occurred at a medical facility, and there was an altercation with a medical professional instead of, or in addition to, a law enforcement officer. A coordinator described:

I just believe that the person probably may be in psychosis or just having a moment. And I believe that, you know, once law enforcement is called, you know, it, the situation you know, may not be able to be diffused, and then I think that leads to an assault, like, say, if the officer tries to arrest the person. Then, you know, the person that's getting arrested, they may not, like, really understand, or, you know, want to know why they're being arrested, and I think that kind of results in the assault and battery on the law enforcement.

Most coordinators also emphasised that participation is contingent on victim approval. In assault‐on‐law‐enforcement cases, they ask the officers for approval. One coordinator stated they ‘haven't come across an officer that did not want the person to be in the docket’. Coordinators described overwhelming victim support for docket participation.

#### 3.2.2 Legal Requirements of Docket Inclusion

The coordinators described how alleged offences impact docket eligibility. All dockets reduce or drop charges upon completion; however, there is a difference between running a pre‐plea or post‐plea programme. Although two dockets run both tracks, the majority run a post‐plea programme. In both programmes, all defendants have been formally accused of one or multiple offences. In a post‐plea programme, participants must enter a guilty plea, thus officially admitting guilt to the court, before entering the programme. In a pre‐plea programme, participants have not admitted guilt and get to participate in the mental health court without pleading guilty or innocent. The benefit of a pre‐plea programme is that individuals can try the docket, and if it does not work out, they can return to the traditional court process and begin the adjudicatory process anew. If they were to have admitted guilt, as they must in the post‐plea programme, then if they fail in the mental health court, they will return to the traditional court and receive their punishment. They do not retain the right to a trial to determine guilt or innocence because they already pleaded guilty.

There was variation in the charges that dockets will consider for participation. Referrals are considered on a case‐by‐case basis. However, two coordinators said their locality only accepts misdemeanours or felonies amended to misdemeanours. Several localities, however, work primarily with individuals facing violent felony charges. They said individuals facing lengthier punishments have more incentive to take part. One coordinator said a history of violence could be a disqualifier for some but not others. Another described a ‘soft rule where we wouldn't take anybody who had a violent felony, a violent felony conviction within 10 years’, but ‘they’ then got rid of this rule to expand programme access. Another coordinator said, ‘it's very rare that we don't want to deal with somebody because of a criminal history’.

#### 3.2.3 Mental Health and Substance Use Diagnoses

To qualify for a docket at all, individuals must be diagnosed with a serious mental illness, most commonly bipolar disorder or schizophrenia, so this in itself was not raised as a concern. Two coordinators considered that the mental illness diagnosis must be evidenced as connecting to the alleged offence. Debates arise when individuals have a co‐occurring diagnosis of mental illness and substance use disorder and whether the individual is a better fit for drug court or a behavioural health docket. Coordinators commonly described their population as high risk and high need. They rely on a tool provided by the Supreme Court of Virginia to determine risk and need.

The process of joining a docket can be long. The most common referral points are the public defender and prosecutor offices. The docket team then looks at the criminal history and Brief Jail Mental Health Screen tool data. They try to find and understand any links between diagnosis and criminal behaviours. It is common for docket officials to conduct a full biopsychosocial assessment to guide recommendations and management, if relevant. Most coordinators meet with potential participants to discuss the programme handbook and requirements. Some require the individual to observe a docket session before agreeing to participate. Coordinators stressed the importance of individuals fully understanding what participation entails and consequences of noncompliance.

An important caveat that arose frequently during the interviews is that mental disorder diagnoses often do not occur in isolation. Coordinators estimated that 70%–80% of their client population have co‐occurring substance use and mental disorders. The most common substances participants use are marijuana and alcohol, followed by cocaine. This complicates participation in a number of ways. First, docket teams must determine if participants fit better on the behavioural health docket or the drug/recovery court docket in their locality. We followed up by asking how they make that determination, and coordinators described a two‐step process. First, they determine if the index offence as charged is more related to their mental disorder or to the substance use disorder. If the latter, then clearly a drug/recovery court is likely to be best. Secondly, they try to determine which of the disorders is primary—either came first or is most influential on behaviour. They described many of the docket holders as not properly diagnosed and having self‐medicated for years, leading to a substance use dependency. They acknowledge, however, the complexity of understanding these relationships, as few are presenting at the beginning of their troubles. This is where the coordinators and justice system team members rely on their mental health team members for optimising recommendations.

#### 3.2.4 The Power of Prosecutors in Docket Admission Decisions

Coordinators described a tension in how the team members approached participant inclusion decisions. On one hand, they consistently described this as a team process, with all members having a voice in the discussion about who to take on and who to exclude. On the other hand, they considered that prosecutors hold a disproportionate amount of power in making this decision. They said that a defence attorney will not recommend their client join the docket, which is a time commitment of at least a year, if the prosecutor is not willing to drop and/or reduce charges upon completion. Given this reality, most cases referred to the docket are sent to the prosecutor first to see whether they would consider dropping and/or reducing the charges. If they say this is a possibility, only then, in most cases, is the defendant screened and assessed for a mental illness.

Then, too, prosecutors have veto power. The political nature of prosecutors can seep into decisions, especially when the charges relate to violent, sexual and/or firearm offences. One coordinator said:

We have it stated that referrals can come from numerous different sources, from probation, pretrial, the courts, police, etc. But from a practical standpoint, if the Commonwealth is not on board with it, it's not going to go anywhere.

Coordinators also described situations where the inclusion and exclusion criteria changed based upon new prosecutors taking office. One coordinator said they began accepting only felony charges due to a change in their head prosecutor. They said:

So someone might have 15 charges, and they're homeless, they need treatment and services, but they're not violent charges. They're not serious charges on paper. And with the new prosecutor's office, we stopped getting referrals for those types of charges, because they were being dismissed in lower courts. So why would any defence attorney refer their client to a programme that's extremely intensive, if they're going to get the same legal outcome without jumping through all the hoops? You know, I would say because they need the help and they need the services and they're homeless and it's in their best interest. But a defence attorney is not going to view it that way. They're going to view it as no, this is not their express interest and we want we want less supervision and fewer trips to the courthouse, not more. And so that's when we really began to see that shift in the types of referrals that were receiving, receiving more violent charges, only felony charges. And we had a lot of internal conversations as a programme like, Hey, this is what we're seeing now, what are we going to do? Are we going to accept these individuals? If so, what is that going to look like? How do we safely manage them? It's taken a lot of tough conversations on and people have disagreed.

Coordinators expressed fear that prosecutors may be less willing to consider cases involving assault‐on‐law‐enforcement officers. This is even if the individual is otherwise a highly suitable candidate because they do not want to ‘upset’ local law enforcement. One coordinator described an individual who was a perfect candidate for their docket; however, the prosecutor would not offer participation because one of the assaulted officers did not approve. In theory, this prosecutor could have moved forward and admitted that individual onto the docket without officer approval. The assaulted officer may express an opinion, and is even encouraged to do so, and this must be seen to be taken into account, but the officer has no other rights here and the court has full powers to decide in all best interests. In practice, prosecutors do not want to risk upsetting local officers they need to work with and/or do not want political blowback for going against law enforcement wishes. In some ways, this makes the prosecutors subservient to political dynamics and less powerful overall, but the coordinators nevertheless regarded them as holding disproportionate power.

## 4 Discussion

The results of this study demonstrate that a primary offence driving individuals with SMI into the U.S. judiciary is physical contact with law enforcement, which initiates a punitive mandatory punishment. Mental health diversion courts provide an opportunity for defendants to receive both the treatment they need and a reduced punishment; however, political factors can impact decisions regarding which defendants are offered admission onto these diversion courts. Our findings show that Virginia's mental health courts are open to using their powers over a range of offences, including recognising that serious and untreated mental disorder may be a factor in direct physical conflict with law enforcement officers in the community and that, for those capable of making any commitment, the best way of preventing further such offending is to get the offender engaged in treatment. Indeed, such offending, crossing the boundary into felony and thus technically out of scope for these courts, was, by a substantial margin, the most common presentation to the courts. In the qualitative component of the study, management of offending against law enforcement officers emerged as a theme in its own right, but also cross‐cut the other themes of stretching the legal requirements for the docket, the complexity of the mental disorder presentations, commonly with illness in conjunction with substance use disorders, and the power of the prosecutors. A major concern emerging from the coordinators indicated that a combination of politics and defence lawyers formed some barrier to likely optimal practice for the accused and the community alike. Defence lawyers, they thought, were likely to advise their clients against seeking a docket because of the short‐term pain—perhaps a year of full commitment to improving their health and social circumstances—and prosecutors, political appointees, leaning to advise against the community approach under the impression that police and public would not like it. There was also the reality that the prosecutors wield disproportionate power, as most defendants are not screened and assessed for a mental illness and offered participation on a mental health docket until the prosecutor indicates they are willing to drop or reduce the charges upon completion.

Prior studies have found that the nature of criminal charges is the first factor that affects diversion opportunities (Wolff et al. 2011), so it is important to know which charges increase the likelihood of docket acceptance or rejection. We found that in Virginia, the assault‐on‐law‐enforcement charge is the most commonly occurring charge. Given that this is a violent felony charge, this indicates that Virginia's diversion courts are more inclusive than those elsewhere, which tend to take lower‐level offences only (Tsai et al. 2018). Drug courts and veteran courts first began considering only individuals with nonviolent charges, but later iterations moved to being more flexible (Belenko 1998; Flatley et al. 2017; V. A. Hiday and Ray 2010; Saum et al. 2001). Thus, it is hard to see why mental health courts should not consider cases of this nature. This widens the scope of individuals who can receive diversion, increases opportunities for those facing lengthier prison sentences and also, by engaging people in treatment who need that treatment to function socially, may be more effective in protecting the community.

The racial implications of these results cannot be ignored. The American criminal justice system has a long history of disproportionate harms against marginalised communities. Invasive policing practices, such as stop and frisk, were disproportionately levied against Black civilians (Brunson and Miller 2006; Kramer and Remster 2018). Black men were disproportionately incarcerated as imprisonment rates soared in the USA (Alexander 2010; Tucker 2017). As mass surveillance technology used by law enforcement continues to proliferate across American communities, neighbourhoods with higher rates of people of colour and higher rates of poverty are bearing the brunt of surveillance deployment (Keener et al. 2026). The results of this study indicate that Black men participating on Virginia's behavioural health and therapeutic court dockets face the assault‐on‐law‐enforcement charge at the highest rate. If courts are required to stick rigidly to 6‐month mandatory imprisonment after every assault‐on‐law‐enforcement officers, however minor, this merely extends discrimination. Offering the option of a diversion docket may help break that cycle of distrust.

Much of the existing research on mental health diversion courts indicates that they produce positive outcomes, primarily in reducing reoffending and arrest rates (Cosden et al. 2003; Fox et al. 2021; A. Hiday et al. 2015; Jalain et al. 2024; Loong et al. 2019; Moore and Hiday 2006; Rossman et al. 2012; Steadman et al. 2011; Trupin and Richards 2003; Yuan and Capriotti 2019). This makes it even more pressing to decide when defendants are offered an opportunity to take part and when their case is excluded from potential participation. Every decision made by a court team to exclude participation is blocking a pathway towards potential positive outcomes. The decision on whether a locality offers a pre‐plea or post‐plea programme can influence whether defendants even consider joining the docket. Pre‐plea programmes are likely to garner the most interest and be recommended by defence attorneys because if participants fail, they can return to the court process without having yet pleaded guilty. The more types of charges a team is willing to consider at the earliest possible stage, the more individuals will be referred, have the possibility to be accepted on the docket and potentially be diverted from further offending.

The clearest public policy recommendation that follows from our results is the need to change the law regarding the 6‐month mandatory carceral sentence (McCausland and Elders 2020) that accompanies an assault‐on‐law‐enforcement officer conviction in Virginia. The interviews revealed that when participants originally received these charges, they were in a state of crisis, and officers were typically not seriously injured. Without willingness or capacity to consider each individual case on its merits, it will be difficult to make progress. As yet, not every location in Virginia offers this option. Prosecutors and defence lawyers alike should be engaged in extending the availability of this docket and in its further evaluation.

Virginia has considered a policy change that would impact this issue, but it has yet to be signed into law. In the 2026 Virginia General Assembly session, House bill 246 and Senate bill 335 passed the state legislature but was vetoed by the governor. The bill, which passed the legislatures with bipartisan support, would have created an affirmative defence for individuals that assault a protected person, such as a law enforcement officer, but have a mental health disorder or intellectual or developmental disabilities. This would create a misdemeanour option so the court could find an individual guilty of misdemeanour assault, which does not carry a six‐month mandatory minimum punishment, when the assault was related to a mental health disorder or intellectual or developmental disability. Criminal justice reform advocacy groups, such as Justice Forward Virginia, and a coalition of organisations working to help individuals with serious mental illness, intellectual and developmental disabilities or on the autism spectrum, such as The Arc of Virginia, Autism Society of Central Virginia and Decriminalize Developmental Disabilities, have tried to convince the governor to sign the bill (Justice Forward Virginia 2026)

Within this policy debate, it must be understood that when individuals incur these charges because they have an underlying mental illness and were in a state of crisis, prison time will not fix this problem. These individuals will still suffer from serious mental illness, and no amount of potential prison time will deter an individual when they are in a state of crisis and they see law enforcement response as a threat. It is also important to recognise that House Bill 246 and Senate Bill 335 are not a perfect solution because they do not help individuals with serious mental illness to be diagnosed and receive services to prevent them from falling into a state of crisis necessitating a law enforcement response. Future efforts should also be directed at fully funding appropriate community mental health services, available no matter the recipients' income and wealth.

### 4.1 Limitations

There are several limitations in the study. We relied on days and times published online when building the quantitative dataset. We were thus reliant on sources for which we could make only limited checks for accuracy and completeness. The annual Virginia reports combine all assault, battery and wounding charges, limiting our ability to check the data against state reports. Qualitative findings add vital detail to practice, but the resulting thematic model is intended to guide further research rather than suggest a definite approach.

## 5 Conclusions

Little detail has been published about the index charges and their context faced by individuals in mental health courts. In Virginia, the most common charge these individuals face is assault‐on‐law‐enforcement officers. This charge, which technically carries a 6‐month mandatory minimum sentence, appears to disproportionately impact Black men. Typically, according to docket coordinators, individuals were in a state of crisis when interacting with police, and the situation escalated to the point of physical contact, leading to this charge. Court teams, especially the prosecutor and defence lawyers, were seen as making critical decisions about whether individuals were eligible for or appropriately tracked into diversion based on their principles rather than informed by outcomes. Our findings provide a case for review of the legislation underpinning practice and informed dialogue with lawyers about the best interests of their clients.

## Funding

The authors have nothing to report.

## Conflicts of Interest

The authors declare no conflicts of interest.

## Data Availability

The data used in this analysis are available upon request to the authors.

## Appendix A Interview Guide Questions

1. What are your roles and duties on the behavioural health or therapeutic docket?
  - How did you start working on the behavioural health or therapeutic docket?
2. What are the roles of all team members who work on your docket?
3. How are participants referred to your docket?
  - What charges are excluded from participation in the docket? Why do you all exclude those charges?
4. What are the standards you all use to determine who is eligible and who is ineligible for docket participation?
  - Do you all have a threshold in terms of diagnosis participants must receive in order to qualify for the docket?
  - How do you all make decisions regarding who is eligible for the docket?
5. What are the most common criminal charges levied against your docket participants?
  - What are the typical precipitating factors for those charges?
  - How common is the assault‐on‐law‐enforcement charges on your docket?
    - When an assault‐on‐law‐enforcement charges is incurred by your participants, what are the precipitating factors that lead to the charges?
  - Does the victim have to approve before the defendant can participate on the docket? Why or why not?
6. How do you handle violations on the docket?
7. How many phases do you have on your docket?
  - How do participants move through those phases?
  - When is a participant eligible for graduation from the docket?
8. How do you all make decisions on specific policies and procedures utilised by your docket?
  - How often do you change your policies and procedures? What drives those changes?
  - Do you all consult with other therapeutic or behavioural health dockets from around the state? Why or why not?
9. What do you think are the biggest challenges for participants on the docket?
  - How have you all handled the decriminalisation and legalisation of marijuana for your participants?
10. How well do you feel like your team works together?
11. What do you think has been the biggest impact on the community of the docket?
12. Have you all run into resistance from local officials or the local community?
  - If yes, how have you all addressed this resistance?
13. Is there anything you wish you had in terms of support from lawmakers?
14. How do you think your docket will evolve in the coming years?
  - How do you think other dockets will evolve in the coming years?
  - What is your biggest concern going forward for therapeutic and behavioural health dockets?
15. What is something that you wish the public knew or better understood about the behavioural health or therapeutic docket?
16. Is there anything that we have not talked about this morning that you think we should know as we move forward with this study?
